# The effect of butylscopolamine on [^18^F]FDG uptake in the gastrointestinal tract is negligible and regionally variable

**DOI:** 10.1186/s13550-023-01012-2

**Published:** 2023-06-20

**Authors:** Falk Gühne, Ferdinand Ndum, Philipp Seifert, Thomas Winkens, Robert Drescher, Martin Freesmeyer

**Affiliations:** Clinic of Nuclear Medicine, Jena University Hospital, Am Klinikum 1, 07747 Jena, Germany

**Keywords:** FDG-PET/CT, Butylscopolamine, Hyoscine butylbromide, Premedication, Gastrointestinal tract, False-positive findings

## Abstract

**Background:**

Butylscopolamine (or hyoscine butylbromide, trade name Buscopan^®^) is occasionally administered as a premedication to reduce non-specific FDG uptake in the gastrointestinal tract based on its antiperistaltic effect. To date, there are no consistent recommendations for its use. The aim of this study was to quantify the reduction in intestinal and non-intestinal uptake by butylscopolamine administration and to derive relevance for clinical evaluation.

**Results:**

458 patients (PET/CT for lung cancer) were retrospectively reviewed. 218 patients with butylscopolamine and 240 patients without butylscopolamine had comparable characteristics. While the SUV_mean_ in the gullet/stomach and small intestine was significantly reduced with butylscopolamine, the colon and rectum/anus showed no difference. The liver and salivary glands showed a reduced SUV_mean_, while skeletal muscle and blood pool were unaffected. An effect of butylscopolamine was particularly evident in men and patients under 65 years of age. There was no difference in the perceived confidence in the assessment of intestinal findings in the subjective evaluation, although in the butylscopolamine group further diagnostics appeared advisable more frequently.

**Conclusions:**

Butylscopolamine reduces gastrointestinal FDG accumulation only in selected segments and, despite a significant effect, only to a small extent. A general recommendation for the use of butylscopolamine cannot be derived from these results, its use for specific issues could be considered individually.

**Supplementary Information:**

The online version contains supplementary material available at 10.1186/s13550-023-01012-2.

## Background

Positron emission tomography/computed tomography using [^18^F]Fluorodeoxyglucose (FDG-PET/CT) is a well-established diagnostic tool in several oncologic and non-oncologic diseases, exploiting the increased glucose metabolism of pathologies to detect tumor manifestations or inflammation. Among others, lung cancer is a common indication for FDG-PET/CT [1, 2]. False-positive findings are common and may reduce the diagnostic accuracy of these examinations [3–5]. In addition, physiological tracer uptake is visible in organs with high metabolism or due to excretion processes and may mask pathologies. In order to increase the validity, various efforts are made to prepare for the examination, such as nutritional conditioning, physical rest and premedication to induce diuresis [6].

The gastrointestinal tract (GIT) with its several sections, is a site of frequent but highly variable tracer uptake, differing in intensity and configuration. A reason for gastrointestinal glucose metabolism and thus uptake is the contraction of the smooth muscle in the organ walls, although other physiological processes do also have an impact [7]. Incidental findings of unknown malignant potential are common in PET imaging [8]. On the other hand, gastrointestinal pathologies such as benign or malignant neoplasms or gastroenteritis are common. Colorectal cancer is the second most common cancer and cancer-related cause of death in Europe, and the third most common in the USA [9, 10]. Detection of bowel malignancies with FDG-PET/CT is challenging due to variable metabolism and a frequent lack of morphological correlation on CT imaging. On the other hand, the sensitivity of FDG-PET/CT for relevant neoplasms in the colon may be as high as 90% [11]. Endoscopic procedures are a diagnostic gold standard and are able to verify a suspicion, but their application is limited in the small intestine, and sensitivity may be lower in the right colon than in the left colon [12, 13]. Furthermore, invasive diagnostic procedures like gastroscopy and colonoscopy carry a risk of adverse events, cause costs and are sometimes limited by lack of availability [14].

The peripheral muscarinic receptor-binding drug butylscopolamine (or hyoscine butylbromide) has an anti-cholinergic effect in preventing smooth muscle contraction of the gastrointestinal, biliary and genitourinary tracts, and is therefore able to reduce bowel movement or colicky pain. Besides its use in symptomatic relief, butylscopolamine is also used in diagnostic procedures such as MRI of the abdomen and pelvis [15–19]. Different institutions use butylscopolamine (trade name e.g. Buscopan^®^) as premedication before FDG-PET/CT with the aim of preventing physiological bowel uptake by decreasing the tonus of wall musculature [20–22]. For this purpose, butylscopolamine has been used for several years in our tertiary care hospital. Because of possible side effects and the low level of evidence, its use was interrupted to re-evaluate the benefit of butylscopolamine for FDG-PET/CT [23–25].

Physiological tracer uptake within the bowel might simulate or disguise pathological findings, leading to false-positive or false-negative FDG-PET/CT results. To optimize PET diagnostics, the highest possible target uptake and the lowest possible non-specific uptake are desired. The aim of this study was to show whether butylscopolamine relevantly reduces FDG uptake in different segments of the GIT and whether the diagnostic value of the examination can be increased as a result, so that butylscopolamine can be recommended as a premedication before FDG-PET/CT.

## Methods

The retrospective analysis was accomplished for two separate full years, the last full year of standardized butylscopolamine administration and the first full year without standardized butylscopolamine administration. From these periods, 965 patients under investigation for lung cancer who underwent FDG-PET/CT were screened. Lung cancer was chosen because of its high frequency in FDG-PET/CT diagnostics and the rarity of associated gastrointestinal tumor manifestations. In the first group, subjects were excluded if butylscopolamine was not administered (due to contraindications such as glaucoma, benign prostate hyperplasia or allergic reactions). In the second group (no-Buscopan group), patients were excluded if they had received butylscopolamine based on an individual decision. Other exclusion criteria were known neoplastic or inflammatory disease of the GIT, missing clinical information on patient characteristics or technical procedures. In addition, only one PET/CT scan per patient was included, so any additional examinations after the first scan were excluded.

All PET/CT scans were performed according to the same standard operating procedure on the same device (Biograph mCT 40, Siemens Healthineers, Erlangen, Germany). Patients who received the premedication had been slowly administered 20 mg of Buscopan^®^ intravenously, 10 min prior to the injection of the radiopharmaceutical. All patients were required to have fasted for at least 6 h prior to the examination. Weight, height and fasting blood glucose were recorded. A standard dose of 250 MBq FDG was used. Patients in both groups also received 20 mg of furosemide intravenously for premedication. Scans were performed 60–120 min after FDG injection and the patients were scanned from the groin to the base of the skull in seven to nine bed positions, acquired for 2 min each. Identical reconstruction parameters were used (iterative TrueX method, iterations: 3, subsets: 24, FWHM: 5 mm, matrix: 200 × 200).

**Fig. 1:**
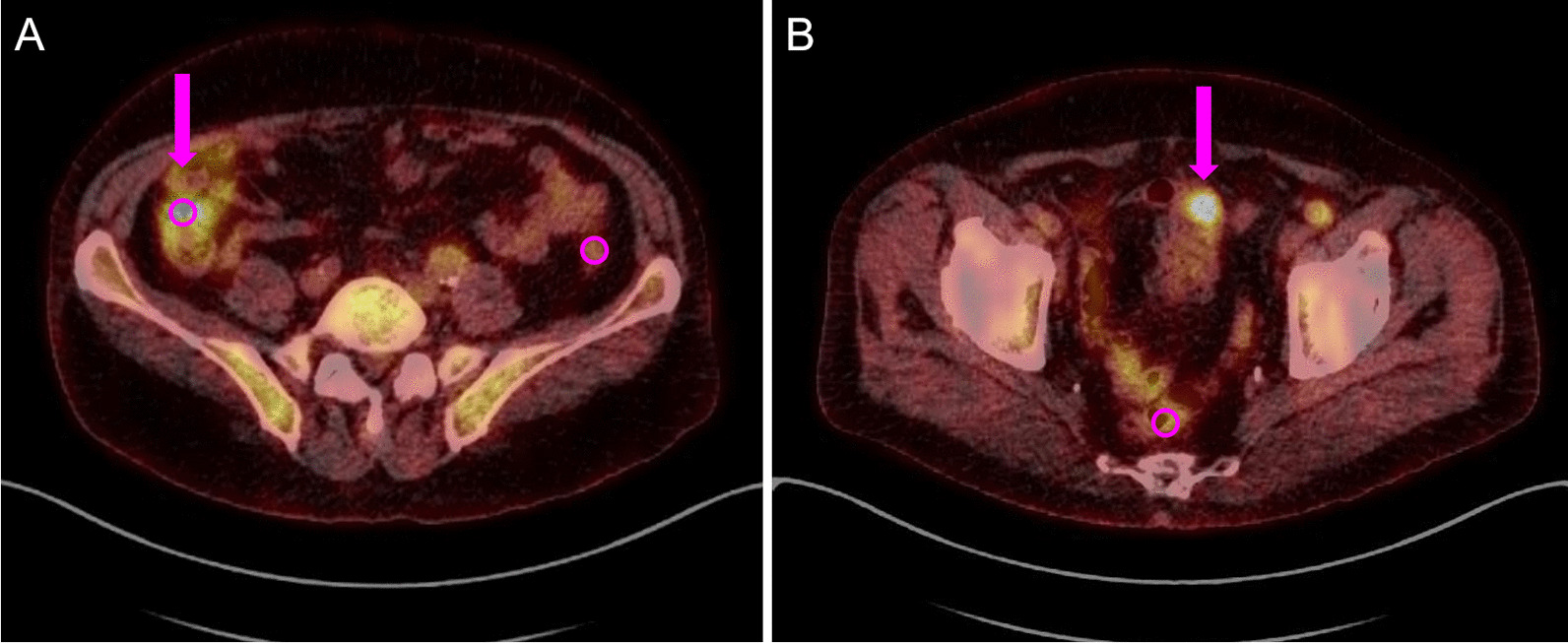
Exemplary depiction of FDG uptake in the bowel of two patients, representing quantitative and subjective assessment. **A** Uptake in the ascending colon (arrow); intensity: moderate, focality: diffuse, probability of being pathologic: probable physiologic, theoretical endoscopy recommendation: no. **B** Uptake in the sigmoid colon (arrow); intensity: high, focality: focal, probability of being pathologic: probable pathologic, theoretical endoscopy recommendation: yes. Standard sized spherical measurement regions (VOI) of SUV_mean_ shown as circles with a diameter of 1.0 cm in ascending colon, descending colon (both **A**) and rectum (**B**)

Data analysis was performed as a second reading, independent of the clinical interpretation of the examinations. One investigator with five years of experience in PET/CT diagnostics assessed the images and was blinded to any knowledge of the written PET/CT reports but was not blinded to the use of butylscopolamine (due to the date of examination). Images were analyzed using the syngo.via multimodality imaging software (Siemens). Quantitative measurements of FDG uptake were performed using the standard uptake value (SUV). SUV_mean_ values were measured in 10 anatomically defined segments of the GIT in a defined spherical volume of interest of 1.0 cm in diameter (volume 0.52 mL) at the location of visibly highest uptake, thus representing a type of SUV_peak_ of the GIT segment. This included the gullet, stomach, duodenum, ileum, colon (with separate measurements for the ascending, transverse, descending and sigmoid colon), rectum and anus. To improve clarity, the 10 segments were grouped into 4 main regions: gullet/stomach, small intestine (duodenum, ileum), colon and rectum/anus. The SUV_mean_ of the GIT regions was calculated as the average of the SUV_mean_ of the included segments. SUV_mean_ of non-GIT organs or compartments was measured in the salivary glands (representative: submandibular glands), the liver, the blood pool (representative: lumen of the ascending aorta), and the skeletal muscle (representative: gluteus maximus muscle). Sex, age, blood sugar and body mass index (BMI) were analyzed separately as confounders of the butylscopolamine effect, subgroups were formed: men versus women; age ≤ 65 years versus age > 65 years, blood sugar ≤ 5.5 mmol/l versus > 5.5 mmol/l; BMI < 25 kg/m^2^ versus BMI ≥ 25 kg/m^2^. Additionally, a subjective assessment of bowel uptake was performed by visual evaluation of intensity (low**,** moderate, high), focality (focal, diffuse) and probability of being pathologic (surely physiologic, probably physiologic, unsure, probably pathologic, surely pathologic), as well as stating the perceived necessity of further diagnostic confirmation (theoretical recommendation for endoscopy) (Fig. 1).

Two-sided unpaired t-tests were used to test for differences between the two groups since the data were largely normally distributed. Box plots were created according to Tukey's definition, with the box ranging from the first quartile to the third quartile and the horizontal line representing the median. The whiskers span 1.5 × the interquartile range from each end of the box. A Pearson's chi-squared test was used to test for independence between categorical variables.

## Results

### Patients

After considering the inclusion and exclusion criteria, 458 patients were included in the study. 240 patients did not receive butylscopolamine (no-Buscopan group) and 218 patients had received butylscopolamine (Buscopan group) as premedication before the FDG-PET/CT. Sex, age, BMI, and blood sugar were not significantly different between groups, while blood sugar tended to be slightly higher in the Buscopan group (Table 1). The scans were performed 59 to 128 min after tracer injection in both groups, with a mean of 76 min (± 14.5) in the group without butylscopolamine and 75 min (± 14.2) in the Buscopan group.

**Table 1:** Patients’ characteristics of total population and the groups of patients with and without application of Buscopan

		Total (n = 458)	Buscopan group (n = 218)	No-Buscopan group (n = 240)	*p* value
Sex (number)	Females	159 (35%)	74 (34%)	85 (35%)	0.74
	Males	299 (65%)	144 (66%)	155 (65%)	
Age (years)	Mean	66.7	66.4	67.0	0.42
	Standard deviation	10.2	9.9	10.5	
	Range	26–89	29–87	26–89	
BMI (kg/m^2^)	Mean	26.0	26.0	25.9	0.68
	Standard deviation	5.4	5.4	5.5	
	Range	15.3–54.5	15.3–48.5	15.8–54.5	
Blood sugar (mmol/l)	Mean	6.0	5.9	6.1	0.09
	Standard deviation	1.4	1.4	1.5	
	Range	3.6–13.0	3.7–13.0	3.6–12.9	

Comparison of groups were accomplished by Chi-squared test for sex and two-sample t-test for age, BMI and blood sugar

### Quantitative comparison

**Fig. 2:**
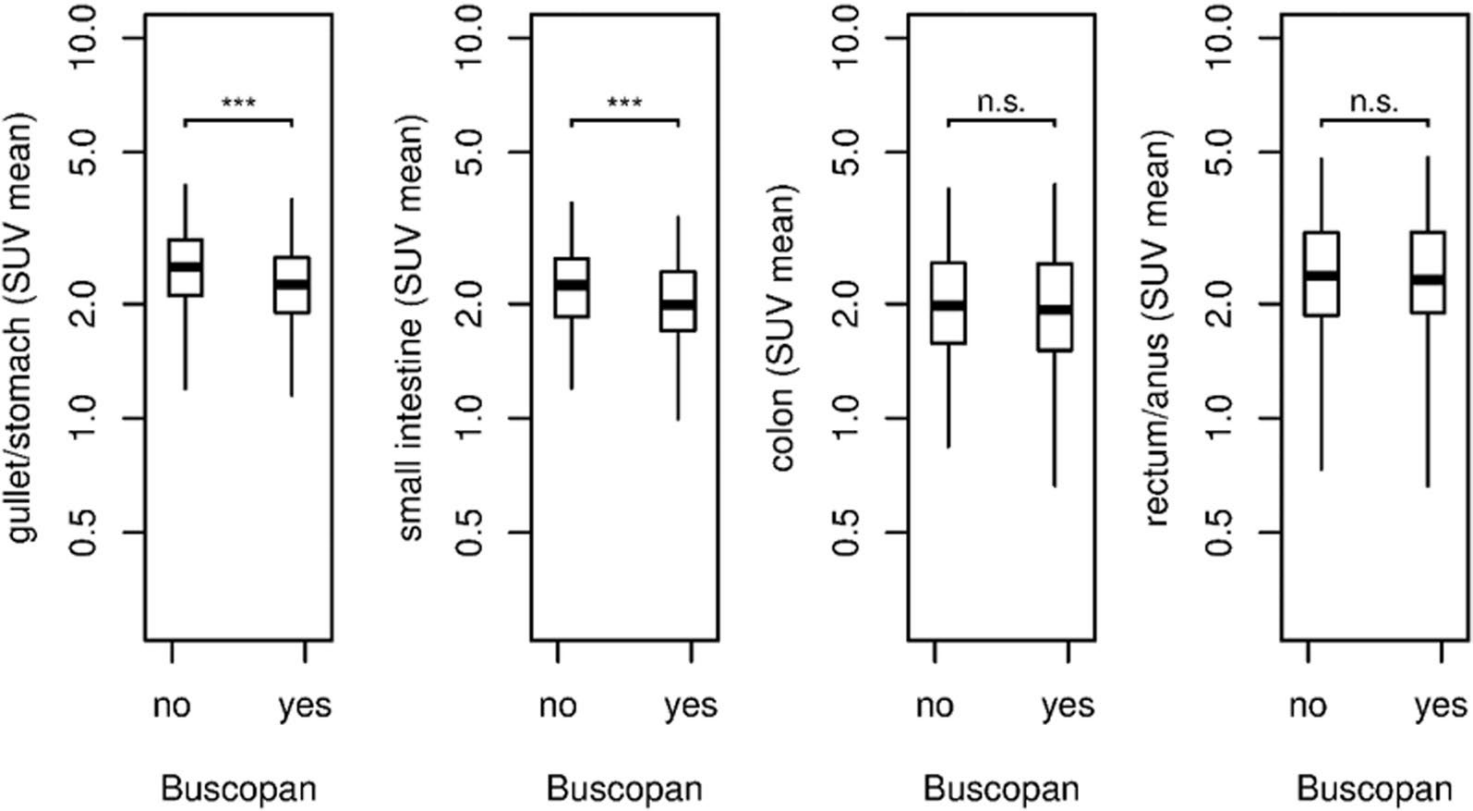
Comparison of SUV_mean_ in the different segments of the GIT between patients with (Buscopan group) and without butylscopolamine premedication (no-Buscopan group). Indication of *p* values: *** = *p *< 0.001, and n.s. =  > 0.05

The segmental comparison of uptake in the GIT between patients having or not having butylscopolamine premedication showed divergent results (Fig. 2). In the group without butylscopolamine, the SUV_mean_ of the gullet and stomach was 2.63 (± 0.90; range 0.9–6.6), while the Buscopan group showed a significantly lower SUV_mean_ of 2.38 (± 0.97; range 0.6–10.5), *p *< 0.001. The SUV_mean_ of the small intestine in the group of patients without butylscopolamine was 2.36 (± 0.92; range 0.9–8.2). In the Buscopan group, the SUV_mean_ was 2.16 (± 0.94; range 0.7–8.6) and therefore significantly lower, *p *< 0.001. The colon showed no significant difference in uptake with a SUV_mean_ of 2.60 (± 1.82, range 0.3–23.9) in the group without butylscopolamine and 2.61 (± 1.91; range 0.3–15.8) in the Buscopan group, *p *= 0.55. As well there was no significant difference in the uptake in the rectum and anus, showing a SUV_mean_ of 2.77 (± 1.64; range 0.3–17.0) in the group without butylscopolamine and a SUV_mean_ of 2.87 (± 1.87; range 0.2–17.9) in the Buscopan group, *p *= 0.89.

**Fig. 3:**
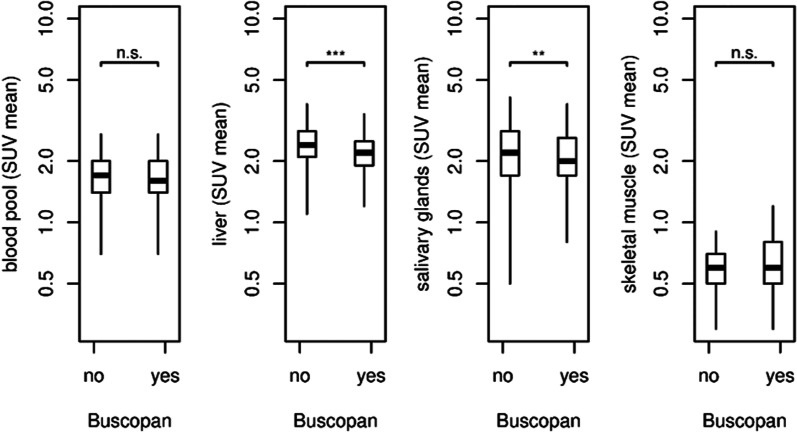
Comparison of SUV_mean_ in the non-GIT-organs and background compartments between patients with (Buscopan group) and without butylscopolamine premedication (no-Buscopan group). Indication of *p* values: *** = *p *< 0.001, ** = *p *< 0.01, and n.s. = *p *> 0.05

Within the non-GIT organs and compartments (Fig. 3), there was no statistically significant difference of the SUV_mean_ in the blood pool between both groups, *p *= 0.19. In the group without butylscopolamine, the SUV_mean_ was 1.72 (± 0.40; range 0.7–3.1), while the Buscopan group showed a SUV_mean_ of 1.67 (± 0.41; range 0.7–3.0). For the salivary glands the group of patients without butylscopolamine showed a SUV_mean_ of 2.35 (± 0.77; range 0.5–5.6), while the Buscopan group showed a significantly lower SUV_mean_ of 2.14 (± 0.65; range 0.8–4.1), *p *= 0.005. In the liver, the SUV_mean_ was 2.45 (± 0.57; range 0.9–7.3) in the group without butylscopolamine and 2.23 (± 0.45; range 1.2–4.0) in the Buscopan group, what was significantly lower, *p *< 0.001. Skeletal muscle showed very similar SUV_mean_ in both groups (0.66 (± 0.21; range 0.3–2.5), and 0.67 (± 0.21; range 0.3–1.5) respectively), without a significant difference, *p *= 0.38.

### Subgroup analysis for butylscopolamine effect

**Fig. 4:**
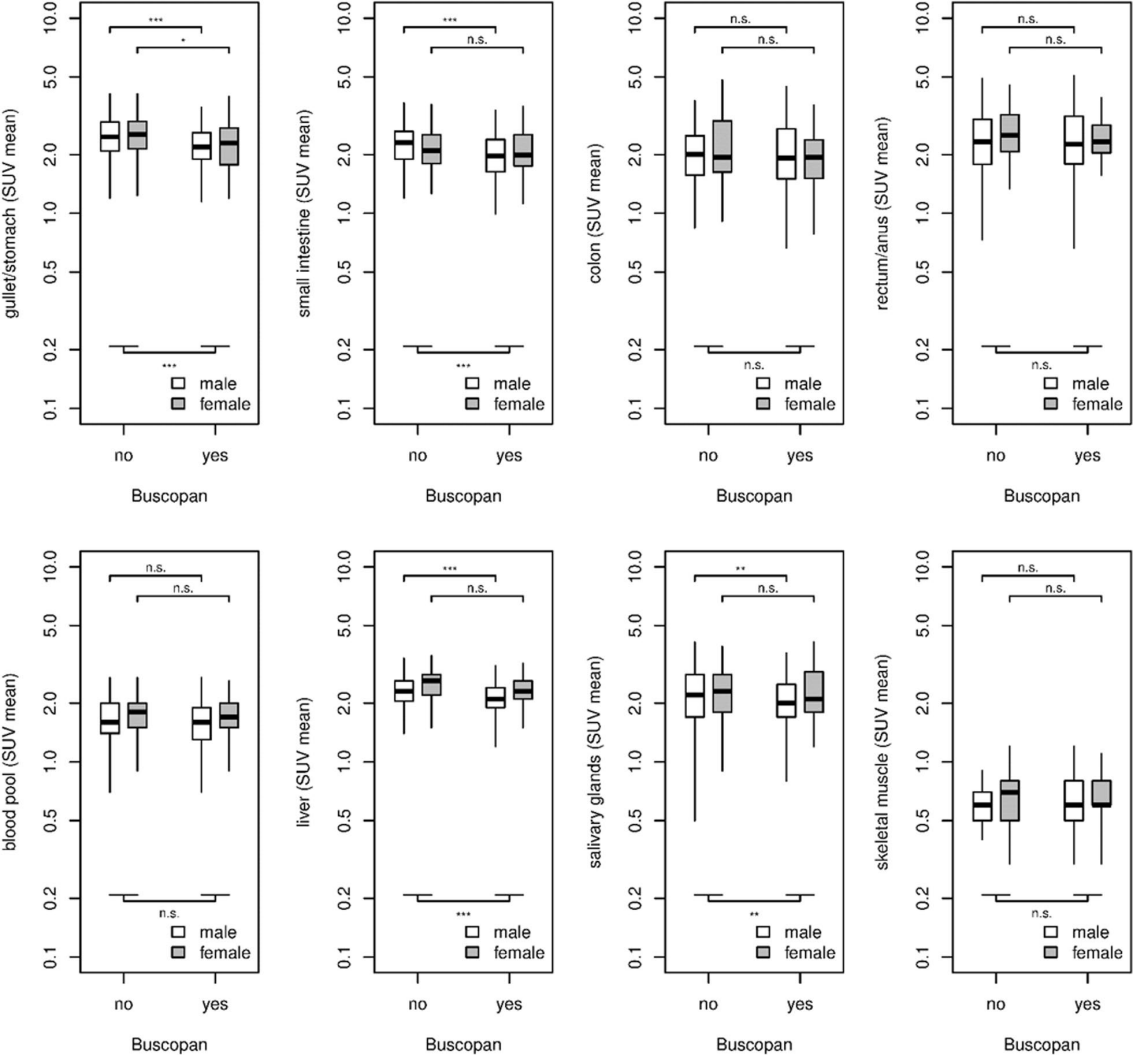
Comparison of SUV_mean_ in the segments of GIT and non-GIT-organs between patients with (Buscopan group) and without butylscopolamine premedication (no-Buscopan group), differentiated by sex. Indication of *p* values: *** = *p *< 0.001, ** = *p *< 0.01, * = *p *< 0.05 and n.s. = *p *> 0.05

Patient sex had a significant influence on uptake in general for the rectum/anus (*p *= 0.004), liver (*p *< 0.001) and salivary glands (*p *= 0.04), showing higher SUV_mean_ in females than in males. All other organs, in particular the GIT segments, did not show any sex-related differences in uptake. Assessing the effect of butylscopolamine with respect to sex revealed a more pronounced decrease of SUV_mean_ under butylscopolamine in men than in women within the following organs: gullet/stomach (men: *p *< 0.001/women: *p *= 0.014), small intestine (men: *p *< 0.001, women: *p *= 0.334), liver (men: *p *< 0.001, women: *p *= 0.061), and salivary glands (men: *p *= 0.003, women: *p *= 0.521). Within the organs that did not show a significant butylscopolamine effect, the differences between the sexes were also insignificant (Fig. 4).

**Fig. 5:**
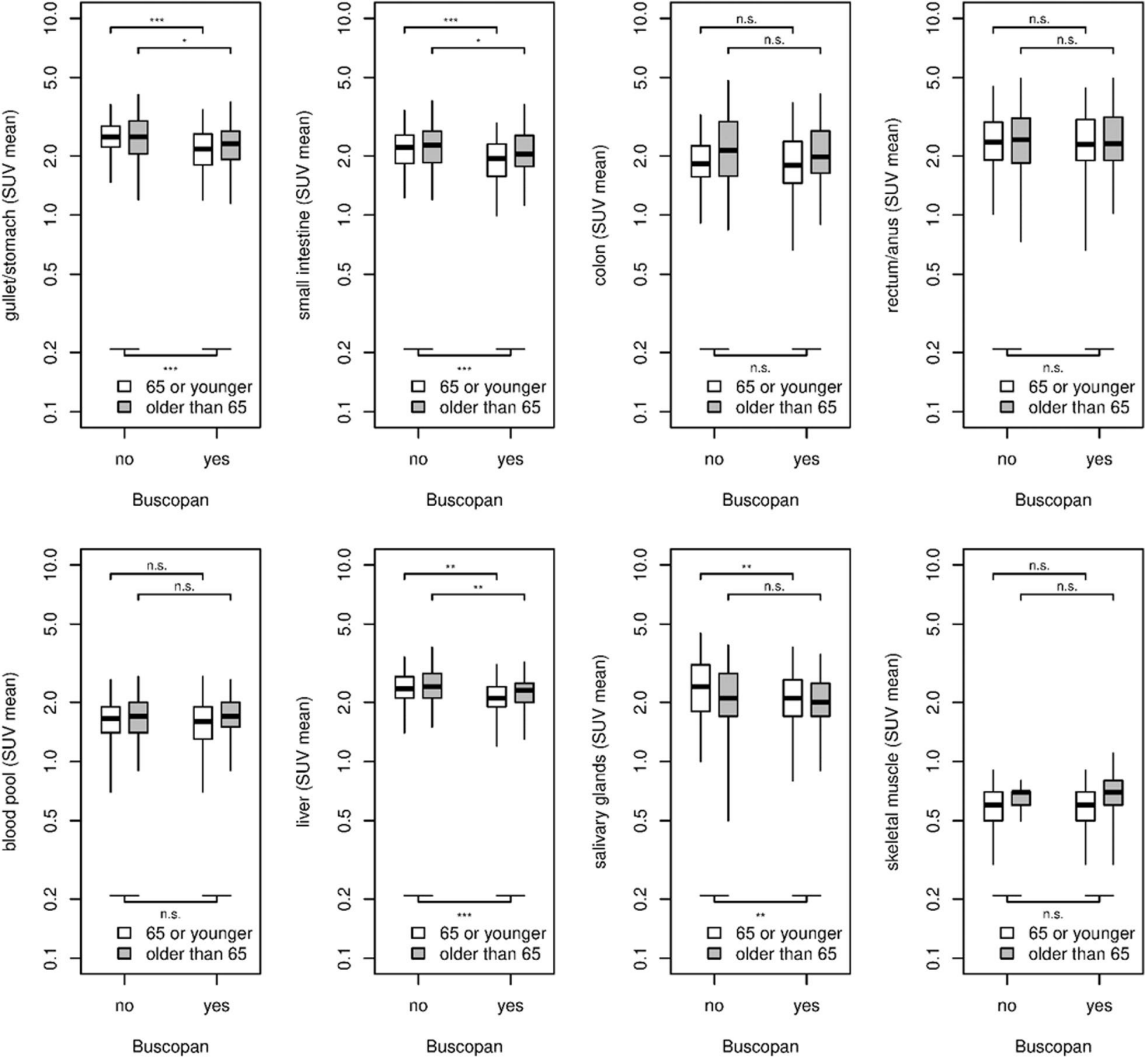
Comparison of SUV_mean_ between patients with (Buscopan group) and without butylscopolamine premedication (no-Buscopan group), differentiated by age. Indication of *p* values: *** = *p *< 0.001, ** = *p *< 0.01, * = *p *< 0.05 and n.s. = *p *> 0.05

For the subgroups of age, patients > 65 years (57% of the total population) had a higher SUV_mean_ than patients aged ≤ 65 years (43% of the total population) in the colon (*p *= 0.002), small intestine (*p *= 0.02), blood pool (*p *= 0.01), liver (*p *= 0.04), and skeletal muscle (*p *< 0.001), but lower SUV_mean_ in the salivary glands (*p *= 0.03). There was no statistically significant difference in SUV_mean_ between younger and older patients in the gullet/stomach (*p *= 0.443) and rectum/anus (*p *= 0.40). Regarding the age-related effect of butylscopolamine, a more pronounced decrease in SUV_mean_ was observed in patients ≤ 65 years of age in the following organs: gullet/stomach (younger: *p *< 0.001/older: *p *= 0.018), small intestine (younger: *p *< 0.001, older: *p *= 0.05), and salivary glands (younger: *p *= 0.005, older: *p *= 0.119) (Fig. 5).

Subjects with blood sugar > 5.5 mmol/l (56% of the total population) showed higher SUV_mean_ values as compared to subjects with lower blood sugar levels in the small intestine (*p *= 0.007), the colon (*p *= 0.004), and blood pool (*p *= 0.03), but lower SUV_mean_ in the salivary glands (*p *< 0.001). Only in the liver, butylscopolamine showed an apparently higher effect in reducing mean SUV_mean_ in patients with blood glucose > 5.5 mmol/l (lower blood sugar: *p *= 0.053/higher blood sugar: *p *= 0.016) (Additional file 1: Fig. S1).

There was a statistically significant difference in SUV_mean_ between BMI subgroups at all measured locations (*p *< 0.001 for each) with higher SUV_mean_ in overweight patients (51% of the total population), except for the salivary glands which showed no BMI-related difference (*p *= 0.09). Only in the salivary glands, the effect of butylscopolamine in reducing the SUV_mean_ appeared to be higher in patients with BMI ≥ 25 kg/m^2^ (lower BMI: *p *= 0.356/higher BMI: *p *= 0.002) (Additional file 2: Fig. S2).

### Visual assessment of GIT

There was no significant difference in the subjectively appraised intensity of bowel uptake between the no-Buscopan and the Buscopan group (*p *= 0.58). Low intestinal uptake was observed in 75 patients (31%) without butylscopolamine and 65 patients (30%) with butylscopolamine, moderate uptake was observed in 89 patients (37%) and 74 patients (34%) and high uptake was seen in 76 patients (32%) and 79 patients (36%), respectively. A higher proportion of patients in the Buscopan group showed focal uptake patterns (70 patients, 32%) than in the group without butylscopolamine (32 patients, 13%). Therefore, a lower proportion of diffuse uptake patterns was seen in the Buscopan group (*p *< 0.001). There was no significant difference between the two groups in the subjective judgement of whether the uptake was pathologic or physiologic (*p *= 0.39). Similar proportions of patients in both groups were rated to have surely physiologic (15% and 14%), probably physiologic (62% and 59%), unsure (12% both), probably pathologic (6% and 11%) and surely pathologic (5% and 4%) bowel uptake. The proportion of patients judged worthy of endoscopic clarification was higher in the Buscopan group (31 patients, 14%) than in the group without butylscopolamine (15 patients, 6%), with a statistically significant difference (*p *= 0.007).

## Discussion

### Appraisal of results

Significant reductions in tracer uptake were quantified in the gullet/stomach and small intestine, but not in the colon and rectum/anus. Even in the first-mentioned segments, the relative reduction under use of butylscopolamine was averagely lower than 10%, while the uptake varied many-fold within the groups, diminishing the relevance of the decrease due to the premedication. The standard deviation was therefore high and did not differ relevantly between patients with and without butylscopolamine, so that no effect on homogenization or narrowing of tracer uptake could be shown. The frequency of incidentally detected malignancies has been described to be higher in the colon and the rectum than in the gullet, stomach, or small intestine [9, 10]. For this reason, a reduction in physiological uptake in the colorectal segments would be of greater impact. Also, the influence of butylscopolamine on bowel uptake may be less because other factors besides peristalsis, such as activation of lymphatic tissue, gut microbiota, swallowed secretion or exfoliated epithelium which could also increase bowel uptake [5, 26]. The intestinal wall musculature appears to have a greater impact on overall glucose metabolism in the gullet, stomach and small intestine, resulting in a more efficient effect of butylscopolamine in reducing the uptake in this region. Additionally, within the stomach the anticholinergic inhibition on parietal cells and principal cells may also reduce the glucose metabolism [27].

The data showed that butylscopolamine does not affect the uptake of non-specific tissue compartments like blood or skeletal muscle. As these serve as a background measures for the visual detection of areas with elevated uptake, the reduction of bowel uptake may be relevant in terms of target/background ratio. In contrast, the salivary glands and the liver demonstrated a significant decrease in uptake with the use of butylscopolamine, probably due to its anticholinergic effect on the muscarinic tissues of the glandular ducts and the biliary system [28]. Thus, the biological effect of the premedication could also be verified and quantified by PET in non-GIT organs. Other organs with muscarinic acetylcholine receptors like inner ocular muscles, smooth muscles of the urogenital system or perspiratory glands can hardly be visualized with PET because of their small size or the retention of tracer-containing urine [28]. Because of the likewise reduction of uptake in the liver by butylscopolamine, a bowel-to-liver ratio, as it has been performed in former studies for comparison, may be ineligible [20, 22].

Previous evidence on the usefulness of premedication with butylscopolamine in PET/CT diagnostics is limited and results have been inconsistent. Former studies indicating a relevant reduction in tracer uptake in the GIT were assessed using PET rather than PET/CT, and the intensity of uptake was primarily determined visually rather than with SUV measurements [20, 22]. A study performed in rats with quantitative measurements on excised gastrointestinal tissue showed, analogous to our results, no significant reduction of FDG uptake by administration of butylscopolamine, but an effect when omeprazole was used [29]. In addition, other options to optimize bowel uptake, such as oral contrast, metformin discontinuation or dual time-point scanning have been discussed [30–33]. According to other authors, SUV normalization methods are important for monitoring intestinal uptake because of their dependence on patient parameters. For this reason, we also investigated the recommended SUV corrected for lean body mass (SUL) [21, 34]. No differences were found between SUL_mean_ and SUV_mean_ regarding the butylscopolamine effect. The significantly higher SUV (butylscopolamine independent) of overweight patients in our data could be resolved by calculating the SUL. The overestimation of SUV by the body weight adjusted determination method is due to a higher proportion of body fat. Until today, no other study investigated the influence of patient characteristics on the impact of butylscopolamine premedication. To evaluate a potential subgroup with the highest benefit for butylscopolamine use, we examined the dependence of sex, age, blood sugar levels, and BMI. The reduction in uptake was especially evident in men and in patients younger than 65 years, whereas no additional effect could be demonstrated in GIT segments other than gullet/stomach and small intestine. Therefore, the value appears to be limited even for subgroups of patients. The proportion of wall tone-associated and muscarinic-mediated uptake in the intestine could thus be lower in women and elderly patients.

The shift from diffuse appearing to focal appearing uptake by using butylscopolamine may have been caused by a reduction of physiologic uptake, revealing pre-existing focal findings. This probably led to a higher number of further diagnostic clarifications being deemed necessary. On the other hand, the proportion of intestinal uptake subjectively assessed to be pathological did not change, nor did the reader’s visual impression. It remains unclear if a higher number of endoscopies would have been clinically indicated to detect existing pathologies, as there is no evidence to compare with. The proportion of incidental findings in FDG-PET/CT is high, but the rate of secondary malignancies is considerably low between 1 and 4% [35–37]. In contrast, other analyses of small populations have shown a high proportion of 48 to 91% of incidental bowel findings with histologic correlation have proven to be true-positive [38–42]. Although the assessment of malignancy risk is subjective and recommendations from PET/CT reports have often been neglected in routine clinical practice, further diagnostic verification of suspicious GIT findings should be attempted.

### Limitations and method

The study has a retrospective design and shows an interindividual comparison. However, the two groups have equivalent patient characteristics and a period of a whole year was chosen for each group to exclude seasonal or dietary influences. There is reliable comparability between both groups given with regard to the relatively large number of patients examined and an unchanged clinical practice (except for the use of butylscopolamine) during this period. Other influencing factors such as metformin or opioid medication cannot be assessed due to the retrospective design and the lack of documentation in clinical practice. Although usage is not necessarily expected to differ significantly between the two groups, it cannot be guaranteed that the effect seen in the data is not caused by an unnoticed decrease of metformin medication between the years 2017 and 2019. This fact represents a significant limitation, nevertheless, it cannot be concluded that the uptake reduction considered is independent of butylscopolamine premedication, because the increase in intestinal uptake under metformin relates in particular to the colon and rectum, for which no difference between the both groups was found in our analysis [43, 44]. A diagnostic gold standard for comparison of PET/CT findings is missing, because an endoscopic evaluation of the entire GIT is not viable for every patient. The theoretical recommendation of endoscopy was limited to the study and therefore not transferred to the treating physician. Even real recommendations were often disregarded or results of further investigations remain unknown. Therefore, the sensitivity or specificity of incidental PET/CT findings in the GIT cannot be calculated. Furthermore, the subjective assessment was performed by a single investigator and the use of butylscopolamine was not blinded. For this reason, these data can only serve as a visual impression to estimate the relevance of the quantitative changes. The transferability of the results from patients with lung cancer to other diseases remains uncertain. However, since it can be assumed that the potential findings in the bowel are incidental, there is not necessarily a dependence on the primary disease. The quantitative approach and the differentiation of GIT segments represents the novelty of this investigation compared to former studies evaluating the influence of butylscopolamine on FDG-PET/CT.

## Conclusion

Even in the era of hybrid imaging, FDG uptake within the bowel remains diagnostically challenging and the avoidance of false-positive or physiological findings on PET/CT is desirable. The spasmolytic drug butylscopolamine is able to reduce the glucose metabolism of the GIT, but only in single segments such as the gullet, stomach and small intestine, and especially in subgroups of patients such as men and under-65-year-olds. The quantitative decrease in uptake is marginal and the visual impression is largely unchanged. Whether more secondary malignancies could be found incidentally remains unclear. In conclusion, the general application of butylscopolamine as premedication for FDG-PET/CT cannot be recommended. Nevertheless, specific issues or patient subgroups may benefit from its use, which remains to be considered.

## Supplementary Information

**Additional file 1: Fig. S1.** Comparison of SUV_mean_ between patients with (Buscopan group) and without butylscopolamine premedication (no-Buscopan group), differentiated by blood sugar. Low blood sugar denotes a level > 5.5 mmol/l. Indication of *p* values: *** = *p* < 0.001, ** = *p* < 0.01, * = *p* < 0.05 and n.s. = *p* > 0.05.

**Additional file 2: Fig. S2.** Comparison of SUV_mean_ between patients with (Buscopan group) and without butylscopolamine premedication (no-Buscopan group), differentiated by BMI. Normal weight denotes a BMI < 25 kg/m^2^, overweight a BMI ≥ 25 kg/m^2^. Indication of *p* values: *** = *p* < 0.001, ** = *p* < 0.01, * = *p* < 0.05 and n.s. = *p* > 0.05.

## Data Availability

The datasets analysed during the current study are available from the corresponding author on reasonable request.

## Abbreviations

BMI: Body mass index
CT: Computed tomography
FDG: [^18^F]Fluorodeoxyglucose
GIT: Gastrointestinal tract
PET: Positron emission tomography
SUV: Standard uptake value
SUL: Standard uptake value corrected for lean body mass
