# Supplementary figures and images for: The effect of butylscopolamine on [18F]FDG uptake in the gastrointestinal tract is negligible and regionally variable
Source: EJNMMI Res. 2023 Jun 20;13:61. doi: 10.1186/s13550-023-01012-2 (PMC10281937; doi:10.1186/s13550-023-01012-2)

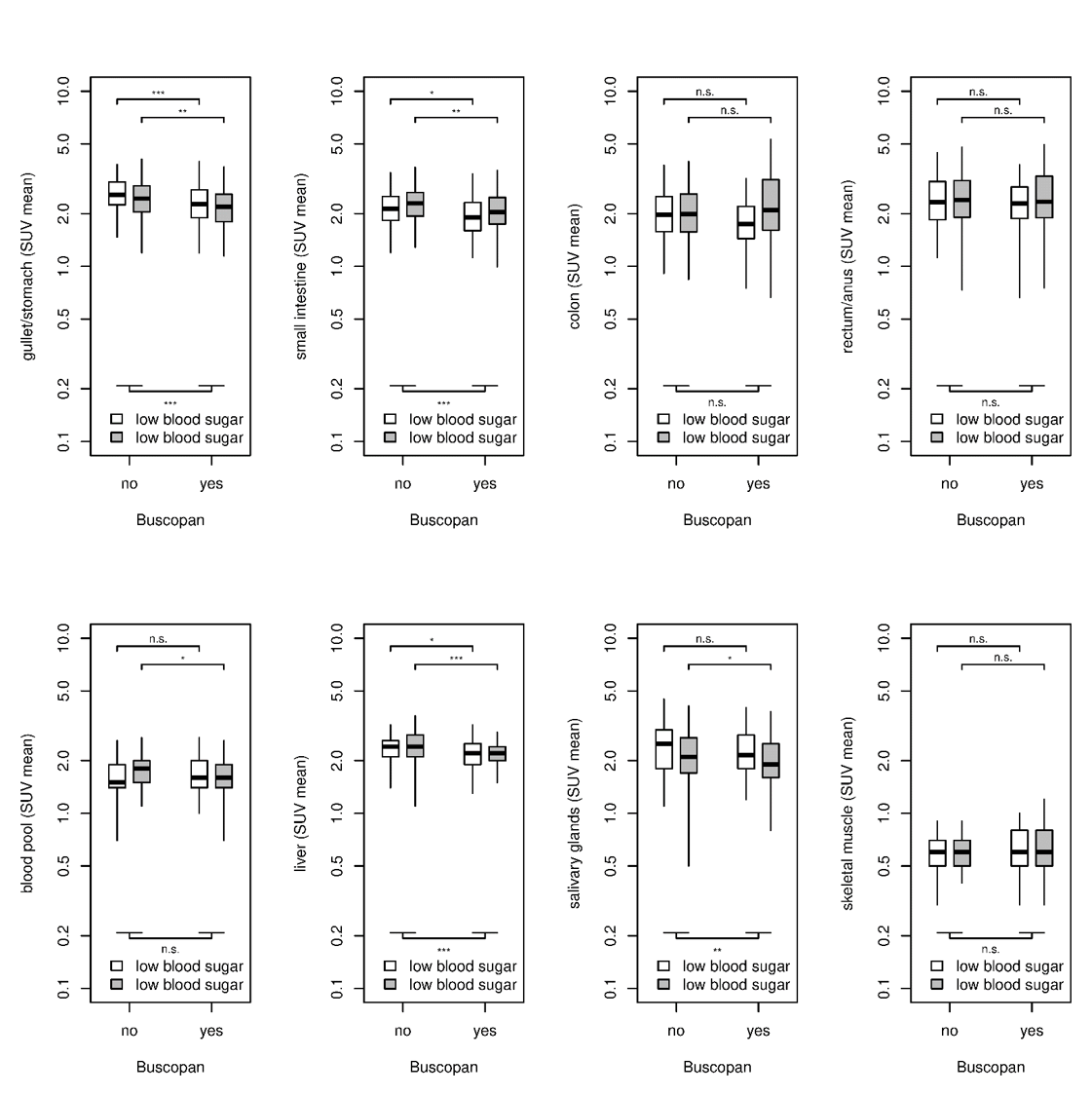

Supplement: Supplementary file 1 — Additional file 1: Fig. S1. Comparison of SUVmean between patients with (Buscopan group) and without butylscopolamine premedication (no-Buscopan group), differentiated by blood sugar. Low blood sugar denotes a level > 5.5 mmol/l. Indication of p values: *** = p < 0.001, ** = p < 0.01, * = p < 0.05 and n.s. = p > 0.05. [file 13550_2023_1012_MOESM1_ESM.tif]

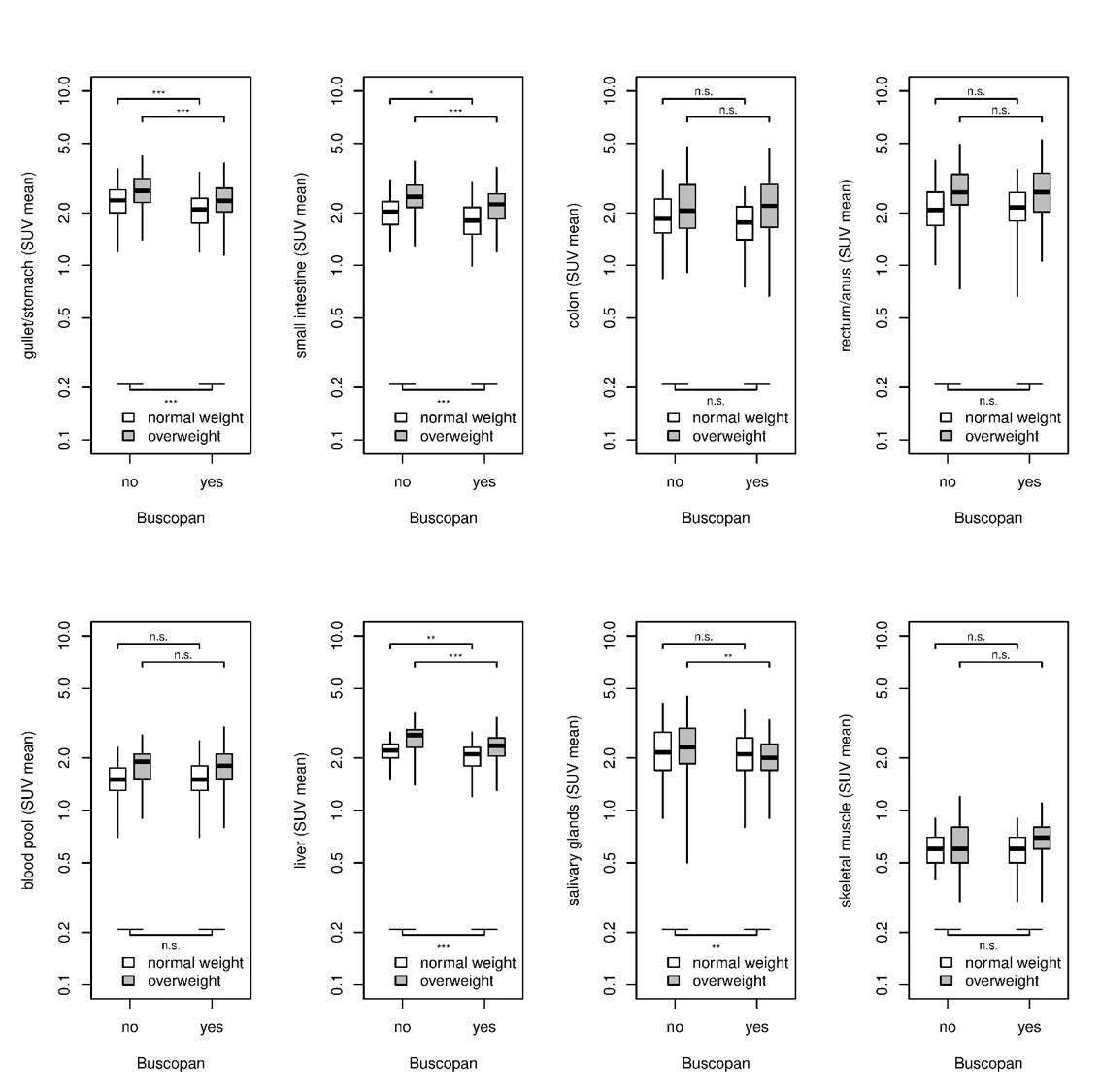

Supplement: Supplementary file 2 — Additional file 2: Fig. S2. Comparison of SUVmean between patients with (Buscopan group) and without butylscopolamine premedication (no-Buscopan group), differentiated by BMI. Normal weight denotes a BMI < 25 kg/m2, overweight a BMI ≥ 25 kg/m2. Indication of p values: *** = p < 0.001, ** = p < 0.01, * = p < 0.05 and n.s. = p > 0.05. [file 13550_2023_1012_MOESM2_ESM.tif]
